# Genome Instability and γH2AX

**DOI:** 10.3390/ijms18091979

**Published:** 2017-09-15

**Authors:** Anastasios Georgoulis, Constantinos E. Vorgias, George P. Chrousos, Emmy P. Rogakou

**Affiliations:** 1 Department of Biochemistry & Molecular Biology, Faculty of Biology, University of Athens, Athens 15784, Greece; tgeorgoulis@med.uoa.gr (A.G.); cvorgias@biol.uoa.gr (C.E.V.); 2 First Department of Pediatrics “Aghia Sophia” Children’s Hospital, School of Medical, University of Athens, Athens 11527, Greece; chrousos@gmail.com; 3 EpigenFocus, Athens 11741, Greece

**Keywords:** γH2AX, H2AX, DNA damage, DDR, DSB, DSB repair, epigenetics, epigenetic biomarker

## Abstract

γH2AX has emerged in the last 20 years as a central player in the DDR (DNA damage response), with specificity for DSBs (double-strand breaks). Upon the generation of DSBs, γ-phosphorylation extends along megabase-long domains in chromatin, both sides of the damage. The significance of this mechanism is of great importance; it depicts a biological amplification mechanism where one DSB induces the γ-phosphorylation of thousands of H2AX molecules along megabaselong domains of chromatin, that are adjusted to the sites of DSBs. A sequential recruitment of signal transduction factors that interact to each other and become activated to further amplify the signal that will travel to the cytoplasm take place on the γ-phosphorylated chromatin. γ-phosphorylation is an early event in the DSB damage response, induced in all phases of the cell cycle, and participates in both DSB repair pathways, the HR (homologous recombination) and NHEJ (non-homologous end joining). Today, numerous studies support the notion that γH2AX functions as a guardian of the genome by preventing misrepaired DSB that increase the mutation load of the cells and may further lead to genome instability and carcinogenesis.

## 1. Genome Instability Is Associated with Diseases and Pathologies

Environmental insults—such as radiation, chemical compounds, etc.—constantly pose a threat to DNA integrity. In addition, cellular processes, such as DNA replication and mitosis, could result in DNA damage if not successfully carried out [1,2].

Although in the long run, mutations are beneficial to evolution as a source of genetic diversity for natural selection, in the short run, mutations have catastrophic consequences to the cells of an organism with repercussions to cancer and degenerative diseases.

To counteract DNA damage, cellular DNA is under the constant surveillance of repair mechanisms, collectively called DDR (DNA damage response). A number of specialized mechanisms have been developed to preserve the genome from different types of mutations, and guarantee faithful chromosome duplication and transmission to the offspring.

Genome instability is defined as an increased rate of genome acquired mutations. The mechanisms leading to genome instability include inherited or acquired defects in the DDR, and more often in DNA DSB (double-strand breaks) repair, DNA replication, cell cycle control, or chromosome segregation [3].

Several diseases and pathologies exhibit genome instability. In multicellular organisms genome instability is central to aging, carcinogenesis and degenerative diseases. It has been demonstrated that aging is associated with the accumulation of somatic mutations. Moreover, the level of genome instability of normal cells is a risk factor for cancer [4]. Another groups of pathologies that exhibit genome instability include neuronal degeneration and immunodeficiency.

In the last decade, the characterization of many proteins involved in DDR has enhanced our understanding in genome instability syndromes and pathologies. Genome instability diseases and pathologies that exhibit mutations in genes encoding DDR proteins are reviewed in Table 1.

Manifestations of genome instability at the molecular level include a variety of DNA alterations, single nucleotide to whole chromosome changes, and typically are subdivided into three categories [5] based on the level of genetic disruption: nucleotide instability (NIN) is characterized by an increased frequency of base substitutions, deletions, and insertions of one or a few nucleotides [6], microsatellite instability (MIN) is the result of defects that lead to the expansion or contraction of short nucleotide repeats called microsatellites, and chromosomal instability (CIN) that leads to changes in chromosome number, alterations in chromosome structure, or aberrations in nuclear architecture [7,8].

## 2. Defective DNA Damage Response Pathways Result to Genomic Instability

Cellular responses to DNA damage, collectively known as DDR, are orchestrated by repair systems that show high specificity. There are three excision repair systems, namely nucleotide excision repair (NER), base excision repair (BER), and DNA mismatch repair (MMR) that correct base mismatches and prevent alterations of microsatellite structure [9]. The DSB repair systems include the non-homologous end joining (NHEJ), that is independent of homology, or utilizes microhomology to join broken ends, predominates in G1, and is error-prone, and the homologous recombination (HR), that promotes accurate repair by copying information from an intact homologous DNA template, and predominates in S/G2 phases [10].

**Figure 1:**
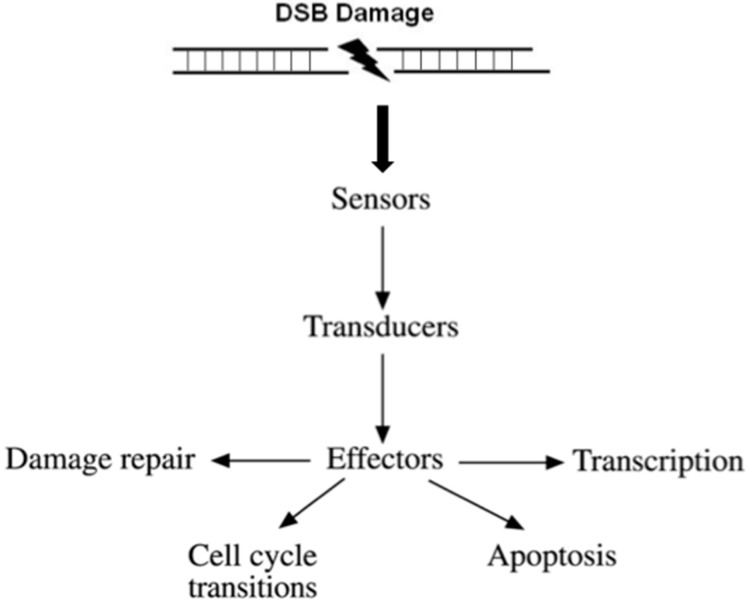
Outline of the DNA damage response signal-transduction pathway. For the purpose of simplicity, the network of interacting pathways are depicted as a linear pathway consisting of sensors, transducers, and effectors.

Conceptually, DDR facilitates a four-step process (Figure 1).

(i) Recognition of the DNA damage. Specialized factors can sense DNA damage and activate the appropriate DNA repair system. These factors are categorized as sensors.
(ii) Generation and amplification of the DNA damage signal. Amplification of the signal is a very critical step in signal transduction as it produces a very large number of activated molecules in order to transduce the signal to the cytoplasm.
(iii) Cross-talk with different cellular pathways to activate effectors; DNA repair effectors, DNA repair induced transcription, and effectors to block cell cycle progression. If DNA damage cannot be repaired in time, DDR activates pathways to drive cells to programmed cell death or senescence, to prevent propagation of damaged DNA into daughter cells.
(iv) Detection of the repaired DNA, and reversal of the previous steps.

## 3. Cellular Processes That Contribute to Genome Instability When DNA Repair Pathways Are Defective

Recent studies have shed light on endogenous sources of DNA damage and chromatin organization that contribute to mutation load, promoting genomic instability and cancer transformation. Here, we mention the most important cellular processes that contribute to genome instability, when DNA repair pathways are defective, that is: telomere maintenance, DNA replication stress, chromosome segregation, epigenetic mechanisms, and RNA processing.

Telomere maintenance: Maintenance of functional telomeres is critical for preventing genome instability. Telomere erosion or uncapping generates catastrophic chromosomal instability through chromosome fusions, followed by bridging during mitosis and further breakage, in breakage-fusion-bridge cycles [11,12].

DNA replication stress: Deregulated DNA replication can derive from replication fork stalling, reversal, and collapse, leading to replication stress. Such replication stress can trigger DNA DSB formation, chromosomal rearrangements, or unscheduled recombination events [13].

Chromosome segregation: Defects in chromosome segregation arise directly through defects in the mitotic checkpoint sister chromatid cohesion, spindle geometry, and spindle dynamics. The outcome of these defects are aberrant chromosome number of affected cells [14].

Epigenetic mechanisms: Epigenetic mechanisms of eukaryotic genomes has been increasingly shown to facilitate DNA repair, to aid maintenance of genomic integrity, and to facilitate stability on DNA sequences. Epigenetic aberrations causing genetic instability are at the root of developmental abnormalities such as immunodeficiency, centromere instability, and cancer [15].

RNA processing: It has been proposed that RNA processing defects destabilize genomes through mutagenic R-loop structures and by altering expression of genes required for genome stability. R-loops are known to play important roles in gene expression regulation by influencing transcription termination, DNA methylation, and chromatin modifications. Thus, the formation of R-loops play a role in genome integrity both by creating a damage-prone sites in the genome and by altering the expression of key genome maintenance proteins [16].

## 4. The Biology of γH2AX

H2AX is a histone mammalian variant that belongs to the H2A family. Histones are proteins that construct the nucleosomes, the basic unit of chromatin. Each nucleosome consists of DNA wrapped around histone molecules; eight histone molecules form a “bead-shape” structure, (two from each of the four core histones (H2A, H2B, H3, and H4), and the linker histone H1 that links the “beads” together. The linker histone, belongs to the H1 histone family, and functions to compact chromatin into higher order structures.

When DSBs are generated into DNA, H2AX becomes rapidly phosphorylated at serine 139. This phosphorylation site in the H2AX carboxyterminal tail is unique among the other members of the H2A histone family. This specific phosphorylation is denoted as “γ-phosphorylation”, and the term “γH2AX” indicates the specific phosphorylation at serine 139 of the histone H2AX [17].

At the H2AX carboxyterminal tail, serine 139 is followed by a glutamine, to form a specific SQ motif. This motif is recognized by kinases that are members of the phosphatidylinositol 3 family (PI3), namely, ATM (ataxia telangiectasia mutated), ATR (ATM and Rad3 related), and DNA-PK (DNA dependent protein kinase) [18,19,20]. ATM is the major kinase to control γ-phosphorylation in human cells [21]. Nevertheless, if ATM activity is diminished, the other kinases take over, indicating overlapping roles between them. It has been demonstrated that ATR is the main kinase to γ-phosphorylate H2AX during replication arrest and under hypoxic conditions [22,23,24]. Other kinases have an important role in γ-phosphorylation in different cellular functions, e.g. during apoptotic DNA fragmentation [25,26].

When DSBs are generated, γ-phosphorylation starts to form almost immediately, and extends both sites of the damage along the chromatin fiber, covering an average distance of megabase long domains in chromatin according the model proposed by W.M. Bonner [18]. However, in some instanses, it has been reported that γH2AX expansion in chromatin fiber spans just a few kilo-bases long [27,28].

There are several scientific questions that need to be addressed regarding the amount of H2AX in different cells. It is known that the percentage of H2AX versus H2A in chromatin is not constant, but spans from 10% to 25% between normal differentiated, as well as cancer cell lines [29,30]. In addition, little is known about the differential distribution of H2AX throughout the genome in different stages of differentiation, or stress conditions. Further research on these topics is needed to shed light on these questions.

The expansion of γ-phosphorylated chromatin along megabase long domains depicts a biological amplification mechanism, where a DSB site is surrounded by thousands of γ-modified nucleosomes. This is the biological basis of a very important practical implication; one DSB can be visualized by immunocytochemistry combined with confocal or epifluorescence microscopy, as the γ-phosphorylation surrounding the DSB site provides the basis to amplify the signal by specific antibodies. Experimental proof of this notion has been provided; during V(D)J recombination, *RAG* (*Recombination-Activating Gene*) mediated cleavage can generate one or two DBSs between immunoglobulins and T-cell receptor loci that can be visualized by microscopy. These results demonstrate that immunocytochemistry with γH2AX specific antibodies is a method with insuperable sensitivity to detect the presence of only one DSB per nucleus [31,32]. Interestingly, in mitotic *Muntiacus muntjak* cells subjected to radiation, γH2AX forms as band-like structures on chromosomes [33], indicating the existence of a higher order chromatin structure that is implicated in the biology of DSBs.

γH2AX is reverted to H2AX after repair restores chromatin integrity and structure. In mammalian cells, several phosphatases are involved in γH2AX dephosphorylation where the phosphatase 2A (PP2A) appears to have a major role [34]. For the elimination of the γ-phosphorylation, another mechanism has also been proposed; the replacement in the nucleosome of the γH2AX by unmodified H2AX molecules [35].

## 5. Specificity of γH2AX for Double-Strand Breaks

It has been well documented that γ-phosphorylation is specific to DSB and does not form in other types of DNA lesions. Experiments with agents that produce other types of DNA damage but DSBs have shown the γH2AX formation is attributed specifically to the DSBs [35,36,37,38].

**Figure 2:**
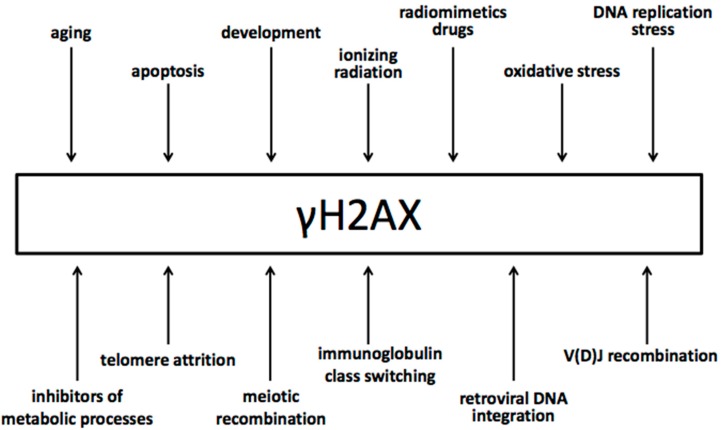
Different sources of DNA double-strand break generation that induce γH2AX.

From the other end, DSBs generated by all different means induce γH2AX formation in cells (Figure 2). DSBs can be generated (i) by environmental agents, such as ionizing radiation, radiomimetic agents, drugs, retroviral integration, etc.; (ii) during cellular metabolic imbalances and malfunctions such as oxidative stress, DNA replication stress, telomere attrition, etc.; (iii) during a variety of cellular processes that include the activity of endonucleases, such as V(D)J recombination, meiotic recombination, apoptosis, etc.

In addition, DNA lesions other than DSBs may be converted into DSBs during subsequent biological processes.

The current dogma is that the generation of a DSB in living eukaryotic cells always induces γH2AX formation, given that these cells have intact the γH2AX pathway.

## 6. γH2AX Mutations as a Factor for Genome Instability

The γ-phosphorylation of the histone H2AX is part of the DDR, specifically the DSB damage early response. γH2AX forms in both HR and NHEJ repair pathways [39]. Nowadays, it is well established that γH2AX is mainly engaged in the signal transduction of the DSB damage response, as it recruits other factors to facilitate signal amplification mechanisms. After activation of ATM, H2AX becomes γ-phosphorylated in seconds, and reaches megabase-long domains in chromatin in 15–30 min. γH2AX recruits Mdc1 (Mediator of DNA Damage Checkpoint 1 protein) to chromatin. Mdc1, in return, facilitates further γ-phosphorylation via feedback loop reactions, possibly by tethering ATM or preventing H2AX dephosphorylation [40]. Working together, Mdc1 and H2AX potentiate the recruitment of many additional factors to the sites of the damage, such as Nbs1 (Nibrin), 53BP1 (p53-binding protein 1), BRCA1 (breast cancer type 1 susceptibility protein), etc.

The DSB damage response, as part of the DDR, is regarded to be a barrier to genome instability and cancer. It has been shown that in early stages of genome malfunction, human cells activate the ATR/ATM-regulated DSB damage response network. This activation is apparent before the occurrence of genomic instability and malignant transformation, and functions to delay or prevent cancer. Mutations that compromise DSB damage response, including defects in the ATM-Chk2 (Checkpoint kinase 2)-p53 pathway, show increased genomic instability and tumor progression [41]. Tissues that bare early precursor lesions, in contrast to the normal tissues, commonly express markers of activated DSB damage response, including phosphorylated ATM and Chk2, p53, and γH2AX. Remarkably, overexpression of different oncogenes that deregulate DNA replication in cultured cells, similar responses were induced [41,42]. In human lung hyperplasias that had no signs of chromosomal instability, signs of DSB damage response were found, including histone H2AX and Chk2 phosphorylation, p53 accumulation, focal staining of 53BP1, and apoptosis. Progression to carcinoma was associated with p53 or 53BP1 inactivation, accompanied by decreased apoptosis [42].

H2AX knockout and knockin models have elucidated our understanding regarding the biological role of the γH2AX. Elimination of the *H2AX* gene, or eradication of the phosphorylation site S129, results in increased sensitivity to DSBs and genomic instability [42,43,44].

Mice that lack the *H2AX* gene are viable, and are characterized by sensitivity to ionizing radiation, growth retardation, premature senescence, immune deficiency, male sterility, impaired cell-cycle arrest, and genomic instability [29,44,45]. Notably, *H2AX^−/−^* mouse embryonic stem cells are more sensitive to DNA damaging factors, and demonstrate severely reduced gene-targeting efficiency [29]. Chromosomal breaks are increased in *H2AX^−/−^* mice cells. In mice that result from cross between *H2AX^−/−^* with *p53^−/−^*, the phenotypic characteristics become more severe, and the mice bear lymphoid and solid tumors [44]. Haploinsufficiency of the *H2AX* gene is shown by the comparison of the number of chromosomal breaks in cells; this number increases between the *H2AX^+/+^* to *H2AX^+/−^* and further to *H2AX^−/−^* mice cells [43,44].

In *H2AX^−/−^* mice, V(D)J recombination products are not affected. Nevertheless, there is a reduction in the absolute number of lymphocytes in *H2AX^−/−^* mice [44], indicating that cells that are unable to repair are eliminated by apoptosis [43]. In *H2AX^−/−^p53^−/−^* mice, where induction of apoptosis is affected, tumorigenesis is increased, attributed either to unrepaired DSBs mediated by the RAG endonuclease, or to spontaneous DSBs [43].

The ability of H2AX to suppress translocations has also been demonstrated in experiments with *Eμ-c-Myc* transgenic mice where a decrease in *H2AX* gene copy number lead to unbalanced clonal and non-clonal translocations in B cell lymphomas of *Eμ-c-Myc^+/−^* mice [46]. The results of these experiments indicate that H2AX haploinsufficiency can cause genomic instability in normal cells and early onset of various tumors including B lymphomas on a p53-deficient background [46].

At the molecular level, loss of the *H2AX* gene does not totally abrogate DSB repair pathways, but it attenuates both HR and NHEJ response in mammals. The critical factors Nbs1, 53BP1, and BRCA1, migrate to the nuclear ionizing radiation-induced foci (IRIF) in *H2AX^−/−^* cells. Nevertheless, despite their initial recruitment to DSBs, they fail to form intense IRIFs [45]. Accordingly, knocking experiments, where the *H2AX* gene is genetically modified to abrogate γ-phosphorylation, have demonstrated similar phenotypes; migration of DSB repair factors was attenuated, and cells exhibited sensitivity to ionizing radiation [43].

The role of γH2AX in concentrating DDR proteins to DSBs in order to form intense IRIF explains why essential regulatory pathways that control the ability of cells to respond to DNA damage are not totally abolished in the absence of H2AX. Cells that have nullified γ-phosphorylation are still viable, nevertheless they show impaired ability to repair DSBs and demonstrate genomic instability.

## 7. Conclusions

Is the past 20 years, γH2AX has been established as a central player in the DDR, with specificity for DSBs. γ-phosphorylation causes an alteration in chromatin structure that facilitates DNA repair signal transduction. γH2AX promotes the transition of chromatin structure to an accessible chromatin to the DSB signal transduction factors, and creates a “docking site” to accelerate their kinetics.

γ-phosphorylation is an early event in the DSB damage response, participates in the two major repair pathways, the HR and NHEJ, and can be induced in all phases of the cell cycle. As γH2AX is a crucial factor of the DSB response, dysfunctions of γH2AX drive towards genomic instability.

In H2AX knockout experiments in mice and human cell lines, it has been shown that H2AX functions as a dosage-dependent suppressor of genomic instability and tumors. γH2AX levels are significantly increased in both precancerous and cancerous lesions, as genomic instability precedes cell transformation. On that basis, several studies have suggested that γH2AX may be used for the diagnosis of cancer development. Translational research on the γH2AX biomarker is very dynamic and is expected to develop further towards these directions.

Human *H2AX* gene maps on chromosome 11, at 11q23. This region exhibits loss of heterozygosity (LOH) or deletion in a large number of human cancers. As many human lymphomas and solid tumors contain deletions of 11q23 on a single allele, loss of a single copy of *H2AX* gene might play a role in unleashing genetic instability in humans.

## Figures and Tables

**Table 1:** Genome instability diseases and pathologies that exhibit mutations in genes encoding DDR proteins. Source: http://repairtoire.genesilico.pl/.

Genome Instability Diseases and Pathologies that Exhibit Mutations in Genes Encoding DDR Proteins		
Disease	Clinical presentation of the disease	Related DDR proteins with impaired function
Ataxia-oculomotor apraxia 1	cerebellar atrophy, ataxia, sensorimotor axonal neuropathy	APTX (aprataxin)
Ataxia telangiectasia	neurodegeneration, immunodeficiency, premature aging, radiation sensitivity, cancer	ATM (ataxia telangiectasia mutated)
Bloom syndrome	immunodeficiency, premature aging, cancer	BLM (Bloom syndrome protein)
Baller-Gerold syndrome	premature fusion of the skull bones and malformations of facial, forearm, and hand bones	RECQL4 (RecQ protein-like 4)
Ataxia-Telangiectasia-like disorder	cerebellar degeneration, radiation sensitivity	MRE11A (double-strand break repair protein MRE11A), ATM
Nijmegen breakage syndrome	microcephaly and mental retardation, immunodeficiency, radiation sensitivity, cancer	NBN (nibrin)
Werner‘s syndrome	immunodeficiency, cancer	WRN (Werner syndrome ATP-dependent helicase)
Rothmund-Thompson syndrome	immunodefiiency, premature aging, cancer	RECQL4
Fanconi anemia	congenital abnormalities, bone-marrow failure, cancer	FANCM (Fanconi anemia group M protein), FANCA, FANCB, FANCC, FANCD2, FANCE, FANCF, FANCG, FANCI, FANCL
Cockayne‘s syndrome	dwarfism, mental retardation, UV light sensitivity	CSA (Cockayne syndrome WD repeat protein CSA), CSB
Xeroderma pigmentosa	UV light sensitivity, skin aging, skin cancer	XPA (Xeroderma pigmentosum group A-complementing protein), XPD, XPB, XPG, POLH (DNA polymerase eta)
Trichothiodystrophy	hair abnormality, mental, and growth retardation	XPB, XPD
Spinocerebellar Ataxia	cerebellar ataxia, axonal neuropathy, muscular atrophy	TDP1 (Tyrosyl-DNA phosphodiesterase 1)
LIG4 syndrome	immunodeficiency and developmental and growth delay	LIG4 (DNA ligase 4)
Progressive external ophthalmoplegia with mitochon	weakness of the external eye muscles and exercise intolerance, cataracts, hearing loss, hypogonadism	POLG (DNA polymerase subunit gamma-1)
Seckel syndrome	growth retardation, microcephaly with mental retardation, a characteristic ‘bird-headed’ facial appearance	ATR (ATM and Rad3 related)
Severe combined immunodeficiency with microcephaly	microcephaly, growth retardation, sensitivity to ionizing radiation	NHEJ1 (Non-Homologous End Joining 1)
Cellular aging	declining ability to respond to mitotic signals and increased homeostatic imbalances	several proteins involved in DNA repair
Cancer	uncontrolled cell proliferation, metastasis	CHEK2 (serine/threonine-protein kinase Chk2 isoform), BRCA1 (breast cancer type 1 susceptibility protein), BRCA2 (breast cancer type 2 susceptibility protein), RAD51 (DNA repair protein RAD51), TP53 (cellular tumor antigen p53 isoform), MLH3 (DNA mismatch repair protein Mlh3), MLH1, MSH2, MSH6, MUTYH (A/G-specific adenine DNA glycosylase), PMS1, PMS2, ALKBH3 (alpha-ketoglutarate-dependent dioxygenase alkB), etc.
